# Characterization of the Therapeutic Effects of Novel Chimeric Antigen Receptor T Cells Targeting CD38 on Multiple Myeloma

**DOI:** 10.3389/fonc.2021.703087

**Published:** 2021-08-26

**Authors:** Xiaorui Li, Yaru Feng, Fengqin Shang, Zhuoying Yu, Tieshan Wang, Jing Zhang, Zhiru Song, Ping Wang, Bingjie Shi, Jianxun Wang

**Affiliations:** ^1^School of Life Sciences, Beijing University of Chinese Medicine, Beijing, China; ^2^Institute of Chinese Medicine, Beijing University of Chinese Medicine, Beijing, China; ^3^School of Traditional Chinese Medicine, Beijing University of Chinese Medicine, Beijing, China; ^4^Shenzhen Research Institute, Beijing University of Chinese Medicine, Shenzhen, China

**Keywords:** chimeric antigen receptor, CAR-T, CD38, multiple myeloma, immunotherapy, xenograft

## Abstract

Multiple myeloma (MM) is a tumor type characterized by the unregulated proliferation of clonal plasma cells in the bone marrow. Immunotherapy based on chimeric antigen receptor T cell (CAR-T) therapy has achieved exciting success in the treatment of hematological malignant tumors. CD38 is highly and evenly expressed in MM and is an attractive target for MM treatment. Here, we successfully constructed two novel second-generation CAR-T cells targeting CD38 by retroviral vector transduction. CD38 CAR-T cells could be activated effectively after stimulation with CD38-positive tumor cells and secrete cytokines such as IFN-γ and TNF-α to promote tumor cell apoptosis in *in vitro* experiments. Real-time fluorescence monitoring experiments, luciferase detection experiments and flow cytometry experiments revealed the efficient and specific killing abilities of CD38 CAR-T cells against CD38-positive tumor cells. The proliferation ability of CD38 CAR-T cells *in vitro* was higher than that of untransduced T cells. Further antitumor experiments *in vivo* showed that CD38 CAR-T cells could be quickly activated to secrete IFN-γ and eliminate tumors. Thus, novel CD38-targeted second-generation CAR-T cells have efficient and specific antitumor activity and may become a novel therapy for the clinical treatment of MM.

## Introduction

Multiple myeloma (MM) is a hematologic malignancy characterized by the uncontrolled expansion of plasma cells in bone marrow (1). The clinical manifestations of disease include signs of end-organ damage such as hypercalcemia, renal insufficiency, anemia and/or osteolytic damage (2, 3). MM accounts for 1% of all cancers, with an incidence of 6-7 cases per 100,000 people, and is the second most common hematological malignant tumor (1, 4, 5). It is estimated that in 2021, a total of 34,920 new cases of the disease will be diagnosed and that 15,600 MM-related deaths will occur in the US (6).

In the past few decades, with the rapid development of new therapeutic approaches, such as proteasome inhibitors (PIs), immunomodulatory drugs (IMiDs), monoclonal antibodies (mABs), autologous stem cell transplantation (ASCT) and histone deacetylase (HDAC) inhibitors, the prognosis and outcome of patients with MM have been significantly improved (7–9). However, MM is still incurable to a large extent and has a high recurrence rate (10, 11). In this regard, new immunotherapies have been developed to combat MM.

Chimeric antigen receptor-engineered T cell (CAR-T cell) therapy has recently emerged with encouraging clinical results in MM (12, 13). The success of CAR-T cells targeting CD19 for chronic/acute lymphoblastic leukemia led to their FDA approval in 2017 (14–16). This has stimulated interest in extending CAR-T cell therapy to MM. CD19 and BCMA are commonly used in clinical trials at present, and novel targets are being developed (17–19). CD38 is highly and uniformly expressed in myeloma cells and is an ideal target antigen for MM (20). Although some CD38 CAR-T cells targeting MM have been reported (20, 21), clinical treatment with more novel CAR-T cells is needed to advance the treatment of hematological cancers. Due to the immunogenicity of the CAR transgene, the host immune response to the mouse-derived single-chain variable region (ScFv) leads to the poor persistence of CAR T cells, and the therapeutic effect is not long lasting (22). In CAR-T cell therapy, humanized ScFv has been applied as a strategy to reduce CAR immunogenicity and subsequently improve efficacy, but it does not effectively prevent the development of anti-IgE reactions that lead to allergic reactions (23, 24). Therefore, the use of human ScFv in the CAR structure may enhance the antitumor activity and persistence of CAR-T cells (23).

Herein, we report two human ScFv-derived, second-generation CAR-T cells targeting CD38. Subsequently, we found that these two kinds of CAR-T cells have notably effective cytokine secretion and specific tumor killing abilities, increased proliferation and expansion abilities *in vitro*, and a very robust xenograft tumor killing ability *in vivo*. Our novel CD38 CAR might be effective for cancer immunotherapy in the clinic. 

## Materials and Methods

### Cells Lines and Cell Culture

The Phoenix-ECO cell line (CLR-3214) and PG13 cell line (TK-NIH3T3) were purchased from the American Tissue Culture Collection (ATCC, Manassas, VA, USA). Both cell lines were cultured in DMEM (Gibco, USA) with 10% fetal bovine serum (FBS, Gibco, USA) and used for retroviral vector packaging. The Daudi cell line (ml-cs-0509) was purchased from the American Tissue Culture Collection (ATCC, Manassas, VA, USA). The RPMI-gfp-luc, Raji-luc, and K562-hBCMA cell lines were kindly provided by Dr. Wu (China Agricultural University). All the tumor cell lines above were maintained in RPMI-1640 medium (Gibco, USA) with 10% FBS.

### CAR Design, Retroviral Vector Production and Detection

The CD38 CAR contains the following components: a signal peptide (SP) and myc taq upstream. The CD38 CAR antigen-targeting regions (ScFvs) contained heavy and light chain variables divided by a (G4S)3 linker, CD8 hinge-transmembrane domain, CD28 coactivation domain and CD3ζ intracellular signaling domain. All these CD38 CAR constructs were packaged into retroviral vectors.

The retroviral vector supernatants were generated by a two-step method using Phoenix-ECO and PG13 retroviral vector packaging cell lines in turn. The copy numbers of the vectors were analyzed by quantitative reverse transcription polymerase chain reaction (RT-qPCR). First, vector RNA was extracted from the vector-containing supernatants (QIAamp^®^Viral RNA Mini Kit, Cat#:52904), and then the vector RNA was reverse-transcribed into cDNA (QuantiNova Reverse Transcription Kit, Cat#:205410). After that, real-time PCR was carried out with the QuantStudio6 Flex system (Applied Biosystems), and the plasmid was continuously diluted to create a standard curve (QuantiNova SYBR ^®^Green PCR Kit, Cat#:75600) with the following formula: (DNA amount (ng)×6.022×10^23^)/(length (bp)×1×10^9^×650). The titer of the recombinant CD38 CAR retroviral vector is reported in transforming units (TU)/mL. This experiment was repeated three times independently. The primer sequences are provided in **Supplement Table 1**.

### Human PBMC Isolation, T Cell Activation, and CAR-T Cell Generation

All blood sampling from the donors was performed after informed consent was obtained, and the protocols were approved by the Beijing University of Chinese Medicine Medical Ethics Committee. Human peripheral blood mononuclear cells (PBMCs) were isolated from healthy human donors by Ficoll density gradient centrifugation (Lymphoprep, Stemcell, Canada). PBMCs were activated with 100 ng/mL soluble anti-CD3, clone OKT3 (Sino Biological, GMP-10977-H001) and 100 U/mL IL-2 (Sino Biological, GMP-11848-HNAE) and then cultured at 37°C in a 5% CO_2_ incubator for 48 hours. T cell growth medium was composed of AIM-V medium (Gibco, USA) with 10% FBS and 100 U/mL IL-2. Activated T cells were transduced using the spin inoculation method (25). The T cell transduction efficiency was measured by flow cytometry (BD LSRFortessa, USA), and the data were analyzed by FlowJo V10. Samples were directly stained with APC-conjugated anti-CD3 (Biolegend, Cat#:300312) antibody and PE-conjugated anti-myc (R&D, Cat#:9E10) antibody. The vector copy number integration-transduced primary T cells were determined by qPCR. The primer sequences are provided in **Supplement Table 1**. Expression of CD38 on CAR-T cell surfaces was stained with APC-conjugated anti-CD38 (Biolegend, Cat#: 102711) antibody and detected by flow cytometry.

### Bioluminescence Imaging-Based Cell Lysis Assays

To determine the lytic ability of CD38 CAR-T cells on CD38-positive tumor cells, CAR-T cells or untransduced T cells (Pan-T) were incubated with RPMI-gfp-luc or Raji-luc cells at different effector: target ratios (E:T ratios) for 12 hours. Luciferase activity was analyzed by a SpectraMax Series Multi-Mode Microplate Reader (model i3x; Molecular Devices). The following formula was used for analysis: % cell lysis = 1 – (experimental lysis – spontaneous lysis) × 100/(maximum lysis – spontaneous lysis). Each experiment was done in triplicate. 

### Fluorescence-Based Assay Using the IncuCyte

RPMI-gfp-luc cells were incubated with CAR-T cells or pan-T cells at an E:T ratio of 1:1. The cells were analyzed by an IncuCyte S3 Live-Cell analysis system (Sartorius, Germany). Images were captured every two hours for a total of 68 hours. Total integrated GFP intensity per well was assessed as a quantitative measure of live, GFP+ tumor cells. The values were normalized to the starting values. Each experiment was done in triplicate. 

### Flow Cytometry-Based Cell Lysis Assays

CAR-T cells or Pan-T cells were incubated with RPMI-gfp-luc, Raji-luc, Daudi cells or K562-hBCMA cells (K562-hBCMA cells expressing BCMA but not CD38 were used as a negative control) at different E:T ratios for 12 hours. Then, the cells were stained with BV421-conjugated anti-CD3, and Annexin V-Alexa Fluor 647 (BioFriend, Cat#: P04D12) staining was used for apoptotic cell detection. Data were collected by flow cytometry and analyzed by FlowJo. The apoptotic rate of tumor cells was calculated as the percentage of CD3-negative and annexin V-positive cells among total cells. Each experiment was done in triplicate. 

### Cytokine Release Assay

To assess cytokine release by CAR-T cells, supernatants were harvested 12 hours after incubation with different types of tumor cells for assessment with a Cytometric Bead Array (CBA, Biolegend, Cat#: B314528) kit following the manufacturer’s protocol. Beads were analyzed by a flow cytometry assay. Each experiment was done in triplicate. 

### Cell Proliferation Assay

CAR-T cells or pan-T cells were labeled with carboxyfluorescein diacetate succinimidyl ester (CFSE, BD, Cat#: 565082) according to the manufacturer’s instructions to track CAR-T cell proliferation. Then, the labeled CAR-T cells were incubated with or without RPMI-gfp-luc cells at an E:T ratio of 1:1 without the addition of exogenous cytokine IL-2 for 72 hours. The proliferation of T cells was assessed by monitoring CFSE dilution. The cells were collected by flow cytometry, and data were analyzed by FlowJo.

### Mouse Xenograft Tumors

NPG/Vst mice are members of the NOD-Prkdc^scid^Il2rg^null^ family. Female NPG/Vst mice with severe combined immunodeficiency were purchased from VITALSTART (Beijing, China). All mice were raised under specific pathogen-free conditions. The animal experiment program was approved by the Biomedical Research Ethics Committee of Beijing University of Chinese Medicine (BUCM-4-2020060101-2015).

NPG mice (6-8 weeks old) were administered intravenous (*i.v.*) inoculations of 2× 10^6^ RPMI-gfp-luc cells. Fourteen days later, the transplanted mice were randomly divided into 4 groups (control group, pan-T group, CD38 CAR21-T group and CD38 CAR22-T group) with 5 mice in each group. On the same day, the mice received *i.v.* injections of 1x10^8^/kg CAR T cells or Pan-T cells. The second injection was given seven days after the first CAR-T cell injection. From the third day after the first CAR-T cell injection (17 days after tumor inoculation), a multifunctional *in vivo* imager (MIIS, Molecular Devices) was used to monitor the tumor load once a week according to the manufacturer’s protocol. On the 16th day after tumor inoculation (the second day after the first injection of CAR-T cells), the peripheral blood of mice was collected, the plasma was separated, and the level of human cytokines was detected by a CBA kit. On the 35th day after tumor inoculation (21 days after CAR-T cell injection), 100 μL peripheral blood of mice was collected, and BV785-conjugated anti-CD3 (Biolegend, Cat#: 300472) staining was performed. Then, the expression of human CD3 in mouse blood was detected to assess the persistence of injected CAR-T cells *in vivo* by flow cytometry. All animals were weighed once a week. The experiments lasted up to 62 days. At the time of death of the mice, blood, liver, bone marrow and lysed erythrocytes were collected. The residual CD38-positive tumor cells were monitored by flow cytometry. 

### Statistical Analysis

The data obtained were analyzed using the GraphPad Prism 8.0 software and expressed as arithmetic the mean ± SEM. Comparisons of two groups or data points were performed by Student’s t-test. All experiments were performed at least 3 times using independent donor cells to ascertain reproducibility. *P*-values < 0.05 were considered statistically significant. 

## Results

### Construction of Second-Generation CD38 CARs and Manufacture of CD38 CAR-T Cells

**Figure 1:**
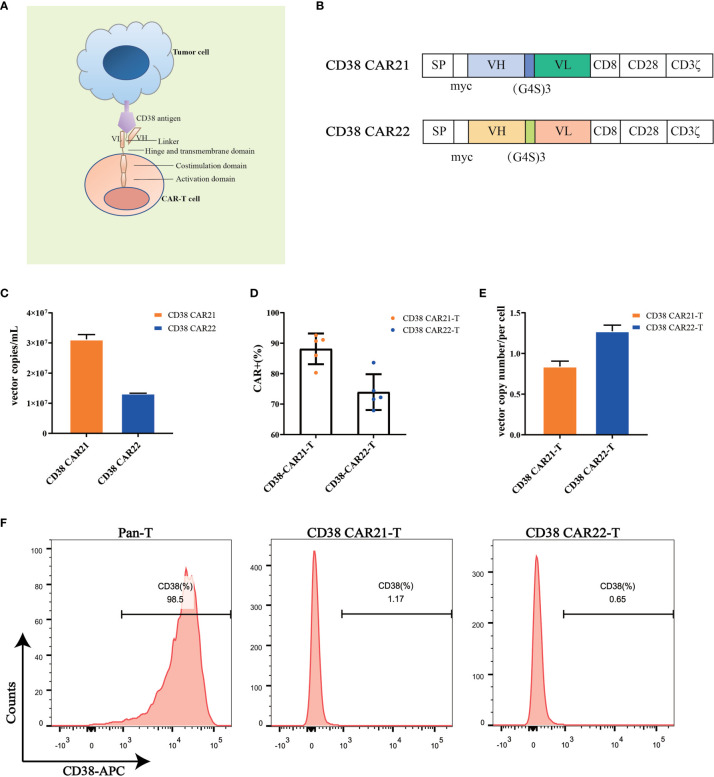
CD38 CAR construct and expression on primary T cells. **(A, B)** The construction of two CD38 CARs. The CD38-ScFv sequences were selected from two different CD38 antibody sequences (CAR21 and CAR22) with the CD8 hinge and transmembrane region, the CD28 cytoplasmic costimulatory region and the CD3ζ signaling domain. **(C)** The copy numbers of supernatant viral vectors produced by the PG13 cell line were detected by RT-qPCR. **(D)** The transduction efficiency of CD38 CAR in primary T cells from different donors (n= 5). The transduction efficiency was defined as the percentage of CD3-positive and myc-positive cells among total live cells. **(E)** The vector copy number of CD38 CAR integration per primary T cell was detected by RT-qPCR. **(F)** The expression of CD38 on the surface of human primary T cells transduced with the CD38 CAR was detected by flow cytometry.

To construct the CD38 CAR, we used the variable heavy and light chain sequences of two different CD38 antibodies as the ScFv sequences, CD8 as the hinge and transmembrane domains, CD28 as the costimulatory domain and CD3ζ as the intracellular activation domain (**Figures 1A, B**). Phoenix-ECO cells and PG13 cells were used to package and produce CD38 CAR retroviral vectors. Vector copy numbers were determined by RT-qPCR, and vector titers were calculated. The results showed that high titers of CD38 CAR retroviral particles were generated (**Figure 1C**). Activated human primary T cells were transduced with CD38 CAR retroviral vectors. The expression of CD38 CAR was detected by flow cytometry. The results showed that the two CD38 CAR retroviral vectors we packaged had high transduction efficiency (**Figures 1D** and **S1A**). The qPCR detection results further confirmed that the two CD38 CARs were integrated into the T cell genome (**Figure 1E**). CD38 expression was seen on the surface of 98.5% of T cells but disappeared after transduction (**Figure 1F**).

### CD38 CAR-T Cells Are Highly Effective and Specific in Killing CD38-Positive Tumor Cells *In Vitro*

**Figure 2:**
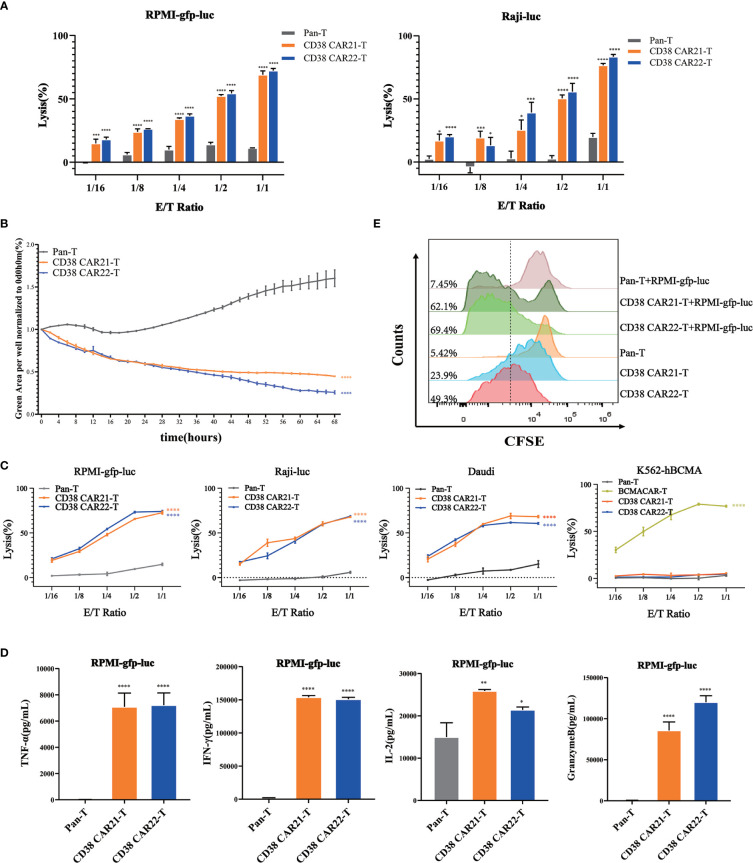
Cytotoxicity and proliferation of CD38 CAR-T cells *in vitro*. **(A)** The ability of CAR-T cells to kill tumor cells was measured by luciferase assay. CD38 CAR-T cells or pan-T cells were incubated with RPMI-gfp-luc cells or Raji-luc cells, and the luciferase signal was determined after 12 hours. **(B)** The ability of CAR-T cells to kill tumor cells was measured by an IncuCyte S3 system. RPMI-gfp-luc cells were incubated with CD38 CAR-T cells or pan-T cells at an E:T ratio of 1:1. Total integrated GFP intensity per well was assessed as a quantitative measure of live, GFP^+^ tumor cells. The values were normalized to the starting values. **(C)** The ability of CD38 CAR-T cells to kill tumor cells was determined by flow cytometry. CD38 CAR-T cells or pan-T cells were incubated with RPMI-gfp-luc cells, Raji-luc cells, Daudi cells or K562-hBCMA cells for 12 hours. **(D)** CD38 CAR-T cells were incubated with RPMI-gfp-luc cells for 12 hours, and the secretion of cytokines was measured with a flow cytometry-based CBA kit. The graph shows the secretion of TNFα, IFN-γ, IL-2 and granzyme B. **(E)** The proliferation of CD38 CAR-T cells. The proliferation ability was measured by the CFSE-based assay. Data were obtained from three replicates and are presented as the mean ± SEM (n=3). *indicates *p* value < 0.05; **< 0.01; ***< 0.005 and ****< 0.001, comparison of two groups was performed by Student’s t-test. TNF-α, tumor necrosis factor α; IFN-γ, interferon γ; IL-2, interleukin 2.

Cytotoxicity is the most important feature of CAR-T cells and directly determines the antitumor activity of CAR-T cells. To determine the cytotoxicity of CD38 CAR-T cells on tumor cells, we established an *in vitro* tumor killing experiment in which CAR-T cells were incubated with different types of CD38-positive tumor cells at different E:T ratios. The results of CD38 expression on the tumor cells are shown in **Figure S1B**. First, the cytolytic effects of CD38 CAR-T cells on tumor cells were analyzed by luciferase assay. We found that compared with Pan-T cells, CD38 CAR-T cells showed a stronger cytolytic effect on RPMI-gfp-luc and Raji-luc cells, and this cytolytic ability was positively correlated with the E:T ratio (**Figure 2A**). Furthermore, we incubated CAR-T cells with RPMI-gfp-luc cells expressing green fluorescent protein at an E:T ratio of 1:1 for real-time monitoring and found that CD38 CAR-T cells had a robust killing effect after 2 hours (**Figure 2B**). These results indicated that our CD38 CAR-T cells have rapid and highly efficient effective antitumor capability.

To confirm the antigen specificity of this antitumor effect of CD38 CAR-T cells, CD38 CAR-T cells were incubated with RPMI-gfp-luc, Raji-luc and Daudi cells expressing CD38 antigen. K562-hBCMA cells that did not express CD38 but expressed BCMA were used as controls. Samples were detected by flow cytometry. The results showed that CD38 CAR-T cells had a robust killing effect on CD38-positive tumor cells but no specific killing effect on CD38-negative K562-hBCMA cells. This result demonstrated the CD38 antigen specificity of the CD38 CAR-T cells against the tumor cells (**Figure 2C**).

The release of cytokines is a marker of CAR-T cell activation. CD38 CAR-T cells were incubated with RPMI-gfp-luc, Raji-luc and Daudi or K562-hBCMA cells, the cell culture medium was harvested, and cytokines such as TNF-α, IFN-γ, IL-2 and granzyme B were measured by a CBA kit. As shown in **Figures 2D** and **S2**, CD38 CAR-T cells secreted large amounts of TNF-α, IFN-γ, IL-2 and granzyme when incubated with CD38-positive tumor cells compared with pan-T cells, and there was no significant change in the killing effect of CD38 CAR-T cells on tumor cells that did not express CD38. The release of these cytokines indicates that CD38 CAR-T cells are effectively activated and further confirms that CD38 CAR-T cells have robust antitumor activity. Moreover, the antitumor activity of CD38 CAR T cells was specific for CD38 antigen.

### CD38 CAR-T Cells Can Proliferate and Expand Rapidly *In Vitro*

The production of a robust and lasting antitumor immune response needs not only to trigger cytotoxicity and the production of cytokines but also to stimulate the proliferation of CAR-T cells. To evaluate the proliferation ability of the two kinds of CD38 CAR-T cells *in vitro*, we divided CD38 CAR-T cells into two groups: single culture and coculture with RPMI-gfp-luc. A CFSE-based assay was used to measure proliferation. The results showed that the green fluorescence signal of the two kinds of CD38 CAR-T cells shifted significantly to the left compared with that of pan-T cells, indicating that the two kinds of CD38 CAR-T cells expanded faster. Moreover, upon stimulation with RPMI-gfp-luc cells, CD38 CAR-T cells showed a better proliferation effect than CAR-T cells cultured alone (**Figure 2E**).

### CD38 CAR-T Cells Efficiently Inhibit Tumor Progression *In Vivo*

**Figure 3:**
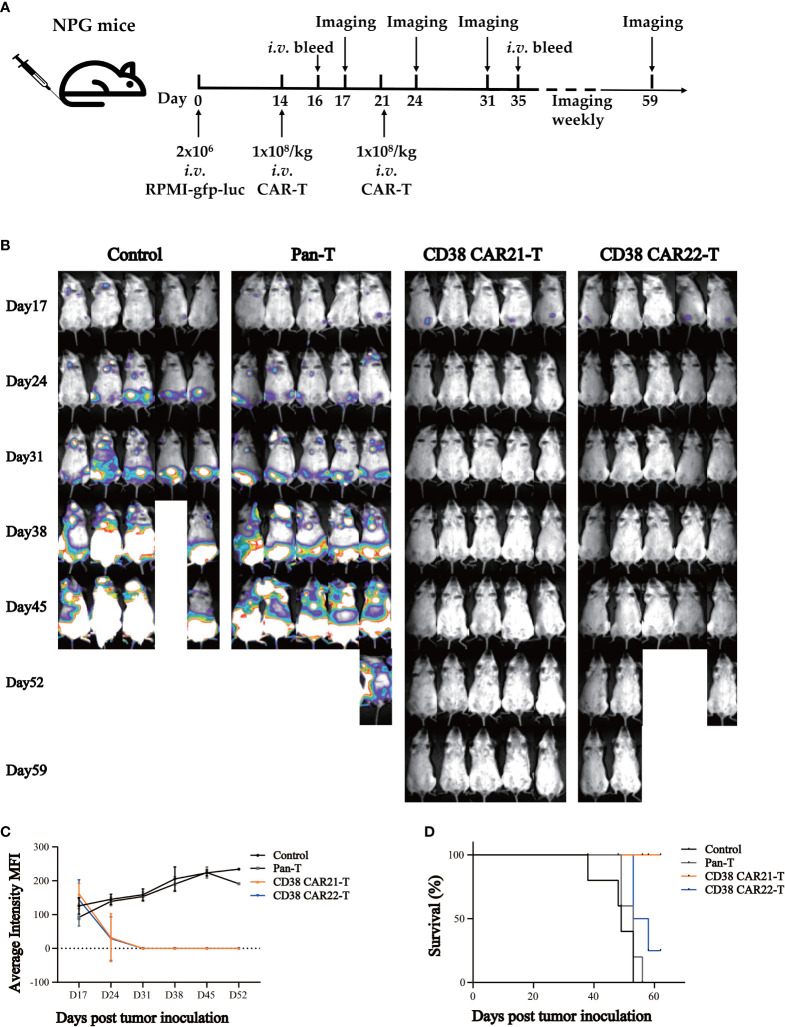
CD38 CAR-T cells exhibited significant antitumor activities in a mouse xenograft tumor model. **(A)** Experimental design indicating tumor cells, timeline, doses, and imaging time points. Mice were randomized after tumor engraftment. T cells from two independent donors were used (n=5). **(B)** Mice were assessed by bioluminescence imaging once a week starting 3 days after the first injection of CAR-T cells. **(C)** Tumor burden was monitored weekly and presented as the average intensity. **(D)** Survival analysis of mice after tumor cell inoculation.n= 5 mice per group.

To evaluate the antitumor effect of CD38 CAR-T cells *in vivo*, we established tumor xenotransplantation models by *i.v.* injection of RPMI-gfp-luc tumor cells. A schema of the experimental protocol for this *in vivo* model is provided in **Figure 3A**. After 14 days, the transplanted mice were randomly divided into 4 groups (**Figure S3A**), and the mice received *i.v.* injections of 1x10^8^/kg CAR-T cells or Pan-T cells. The second injection was given seven days after the first CAR-T cell injection. As shown in **Figures 3B, C**, the results of bioluminescence imaging *in vivo* showed that compared with the control group or Pan-T group, the tumor of the CD38 CAR-T cell groups was cleared completely after the second injection of CD38 CAR-T cells. At the end of the study, the CD38 antigen was virtually undetectable in the blood, liver and bone marrow (**Figure S4**), showing a strong antitumor ability *in vivo* until the end of the experiment, and no recurrence was observed. On the 16th day after tumor cell inoculation (the second day after the first injection of CAR-T cells), we found that the level of human IFN-γ in the blood of mice treated with CD38 CAR-T cells was significantly higher than that of mice in the Pan-T group, which indicated that CD38 CAR-T cells produced more IFN-γ and that CD38 CAR-T cells were effectively activated (**Figure S3B**). At 35 days after tumor inoculation, human T cells were still detected in the blood, and the number in the CAR-T group was higher than that in the control group (**Figure S3C**). This result demonstrated that CD38 CAR-T cell persistence was obviously increased *in vivo* compared with pan-T cell persistence, indicating that CAR-T cells could survive longer *in vivo* and have a more lasting antitumor effect. The survival rate of mice in the CD38 CAR-T groups was significantly higher than that in the control group and the Pan-T group, and the survival rate of CD38 CAR21-T cells was 100% until the end of the experiment (**Figure 3D**). In addition, the weight of mice treated with CD38 CAR-T cells was relatively stable compared with that of the pan-T group (**Figure S3D**).

## Discussion

MM is a malignant tumor mainly characterized by the malignant proliferation of plasma cells in bone marrow that secrete a large amount of monoclonal immunoglobulin (M protein) and eventually leads to organ damage (26, 27). MM is still incurable. Adoptive cellular immunotherapy is a promising method for the treatment of cancer. In recent years, immunotherapy based on CAR T cells has achieved remarkable success in the treatment of malignant hematological tumors, especially lymphoma (26, 28). With the success of CAR-T cells in the treatment of acute lymphoblastic leukemia, research on CAR-T therapy in MM has continuously advanced (8). The human CD38 antigen is a transmembrane glycoprotein that is highly expressed in MM cells. Although CD38 is also expressed on B cells, plasma cells, T cells, NK cells and myeloid progenitor cells, the expression level is much lower than that in MM cells (29). Anti-CD38 monoclonal antibodies such as daratumumab, have been approved by the FDA for the treatment of newly diagnosed MM (30) and relapsed/refractory MM (31). Compared with anti-CD38 monoclonal antibodies, CD38 CAR-T cells have substantially improved specificity and can induce a durable and efficient response (32). Although CD38 CAR-T cells targeting MM have been reported, there are still no approved CD38 CAR-T cell products for clinical use; thus, more novel CD38 CAR-T cell clinical treatments are needed to advance the treatment of hematological cancers. In this study, we demonstrated the great potential of using novel human ScFv-derived CAR-T cells targeting CD38 in the treatment of MM. MM cells express many markers; among them, CD38 molecules are uniformly and highly expressed on the surface of myeloma cells. Recently, many studies have suggested that CD38 may be an ideal target for antigen-specific adoptive cell therapy (20, 33, 34).

CARs mainly comprise single-strand variable regions as signal-binding domains and a linker that connects the heavy (VH) and light chain variable (LH) extracellular domains. In our study, we used a flexible linker (G4S)3 consisting of glycine and serine to increase the flexibility of target cell binding regions (35). At present, clinically used CAR-T cells mostly contain mouse-derived ScFv as the extracellular signal domain, but the host may produce an immune response to mouse-derived ScFv, resulting in a human anti-mouse antibody reaction, thus reducing the efficacy (36, 37). The ScFv sequences of two human CD38 antibodies screened by a previous phage display technique in our laboratory can reduce the immunogenicity of the CAR. The transmembrane region usually comes from CD8α, and the intracellular moiety contains the CD3ζ signaling chain. The costimulatory domain of second-generation CAR cells usually uses CD28 or 4-1BB, and CD28 promotes the secretion of cytokines by CAR-T cells and shows stronger antitumor ability (38–41). Third-generation CARs have two costimulatory domains to promote the proliferation and activation of CAR-T cells (42). At present, second-generation CARs are the most widely used in the clinic and the most effective (3). Therefore, in this experiment, we used the second-generation CAR structure, and the intracellular stimulation we used was CD28. We stably integrated CD38 CARs into the genome of mitotic T cells with a retroviral vector. We found that there was no expression of CD38 on the surface of successfully prepared CD38 CAR-T cells, but it did not affect the function of CD38 CAR-T cells, which was also described in other studies (39, 43). The loss of CD38 may be due to the “self-lysis” of CD38-positive T cell compartments (44).

To determine the antitumor ability of CD38 CAR-T cells *in vitro*, we selected different kinds of CD38-positive cells and used CD38-negative tumor cells as a control. In different experimental studies, we found that CD38 CAR-T cells have an efficient killing effect on CD38-positive tumor cells but not CD38-negative tumor cells. These results indicate that our CD38 CAR-T cells have a specific killing effect on tumor cells. The distribution of CD38 expression is rather extensive, and as such, normal cells expressing CD38 may be lysed by CD38 CAR-T cells; therefore, this situation cannot be ignored. In the cytokine secretion experiment, CD38 CAR-T cells secreted proinflammatory cytokines after being stimulated by tumor cells, which promoted the apoptosis of tumor cells. CD38 CAR-T cells could be activated by CD38-positive tumor cells but not by tumor cells that do not express CD38, which further confirms the killing specificity of this kind of CD38 CAR-T cell. These results are consistent with those of previous studies (34, 44, 45). We found that CAR-T cells proliferated faster than pan-T cells, and under the stimulation of tumor cells, the proliferation ability of CAR-T cells was further enhanced. It is possible to be due to the coactivation domain CD28. CD28 is an important co-stimulatory molecule for T lymphocytes, which could deliver signals that complement T cell receptor (TCR) in both qualitative and quantitative manners, thereby promoting T cell proliferation and survival (46).

Furthermore, we established NPG mouse xenograft tumor models to determine the antitumor ability of CD38 CAR-T cells *in vivo*. We found that IFN-γ cytokine secretion was significantly enhanced and that CD38 CAR-T cells were effectively activated two days after tail vein injection of CD38 CAR-T cells, which indicated strong antitumor ability. Moreover, there was no recurrence of tumors observed during the continuous observation period, and flow cytometry showed that CD38 CAR-T cells could persist in mice. This shows the persistence of the antitumor effect of CD38 CAR-T cells.

## Conclusions

Overall, we successfully constructed second-generation human ScFv-derived CD38 CAR-T cells and assessed the *in vitro* functional activity, such as cytolysis and cytokine release, on CD38-positive tumor cells. These CAR T cells can significantly inhibit xenograft tumor growth *in vivo*, highlighting their antitumor effect. This research provides a solid foundation for further clinical study and illustrates that this CD38 CAR-T therapy has very good clinical potential.

## Data Availability Statement

The raw data supporting the conclusions of this article will be made available by the authors, without undue reservation.

## Ethics Statement

The studies involving human participants were reviewed and approved by Beijing University of Chinese Medicine Medical Ethics Committee. The patients/participants provided their written informed consent to participate in this study. The animal study was reviewed and approved by Biomedical Research Ethics Committee of Beijing University of Chinese Medicine.

## Author Contributions

JW conceived the project and supervised the experiments. XL performed the experiments with the help of YF, FS, ZY, ZS, and BS. XL, TW, and ZY participated in the figure preparation and revised the manuscript. XL wrote the manuscript with revisions by JW and PW. All authors contributed to the article and approved the submitted version.

## Funding

This work was supported by “Double First-Class” start-up funds from Beijing University of Chinese Medicine (to JW; No. 1000041510155).

## Conflict of Interest

The patent application was filed based on the work reported in this manuscript.

The authors declare that the research was conducted in the absence of any commercial or financial relationships that could be construed as a potential conflict of interest.

## Publisher’s Note

All claims expressed in this article are solely those of the authors and do not necessarily represent those of their affiliated organizations, or those of the publisher, the editors and the reviewers. Any product that may be evaluated in this article, or claim that may be made by its manufacturer, is not guaranteed or endorsed by the publisher.
