# Supplementary material for: Characterization of the Therapeutic Effects of Novel Chimeric Antigen Receptor T Cells Targeting CD38 on Multiple Myeloma
Source: Front Oncol. 2021 Aug 26;11:703087. doi: 10.3389/fonc.2021.703087 (PMC8427526; doi:10.3389/fonc.2021.703087)
Supplement: Supplementary file 1 [file DataSheet_1.docx]

Supplementary Material

**
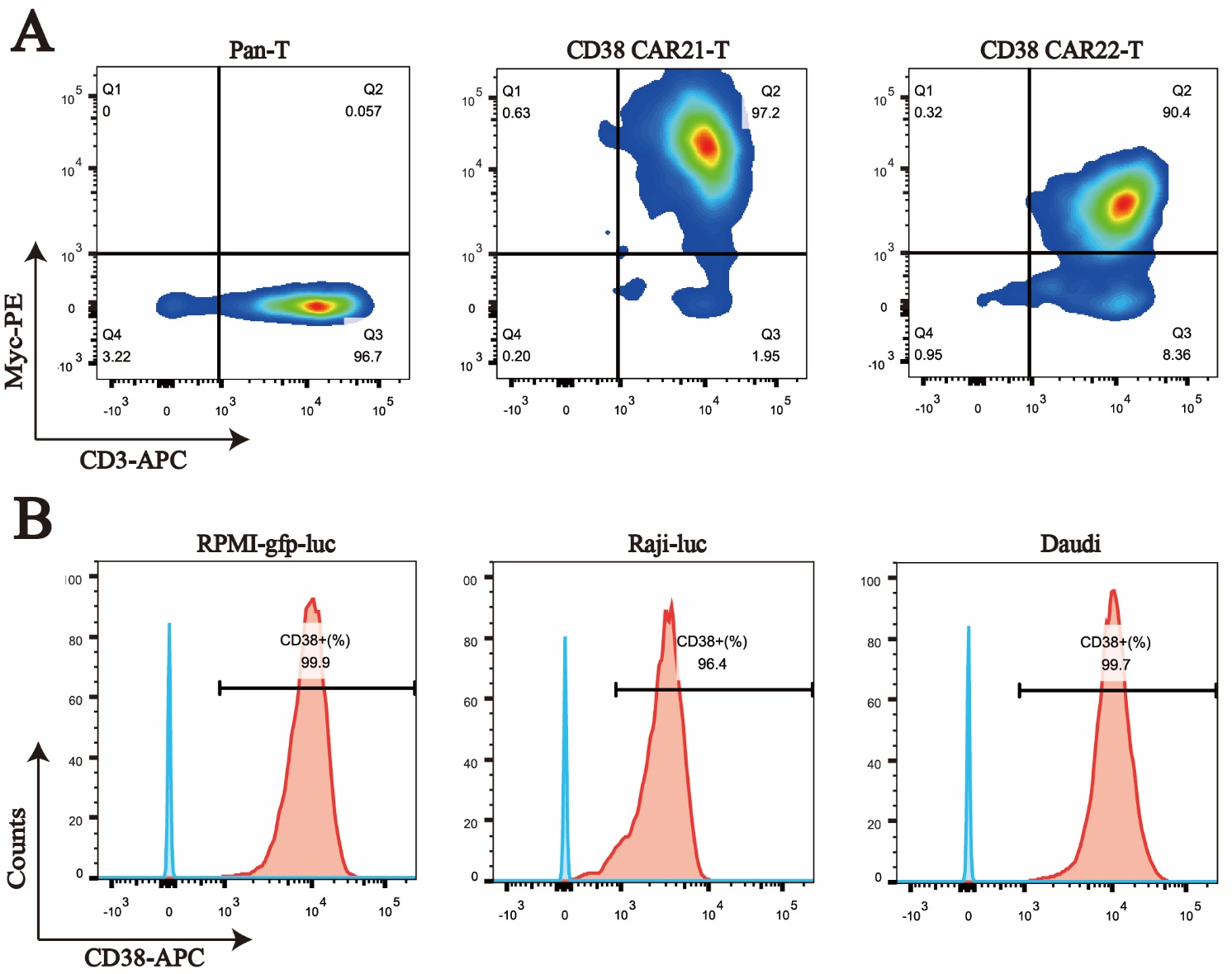
**

**Supplementary Figure 1.** **The transduction efficiency of CD38 CAR and the expression of CD38 antigen on tumor cells.** (A) The transduction efficiency of the CD38 CAR retroviral vector in primary T cells. (B) The expression of CD38 antigen on the surface of different types of tumor cells. K562-hBCMA cells that did not express CD38 were used as a negative control.

**
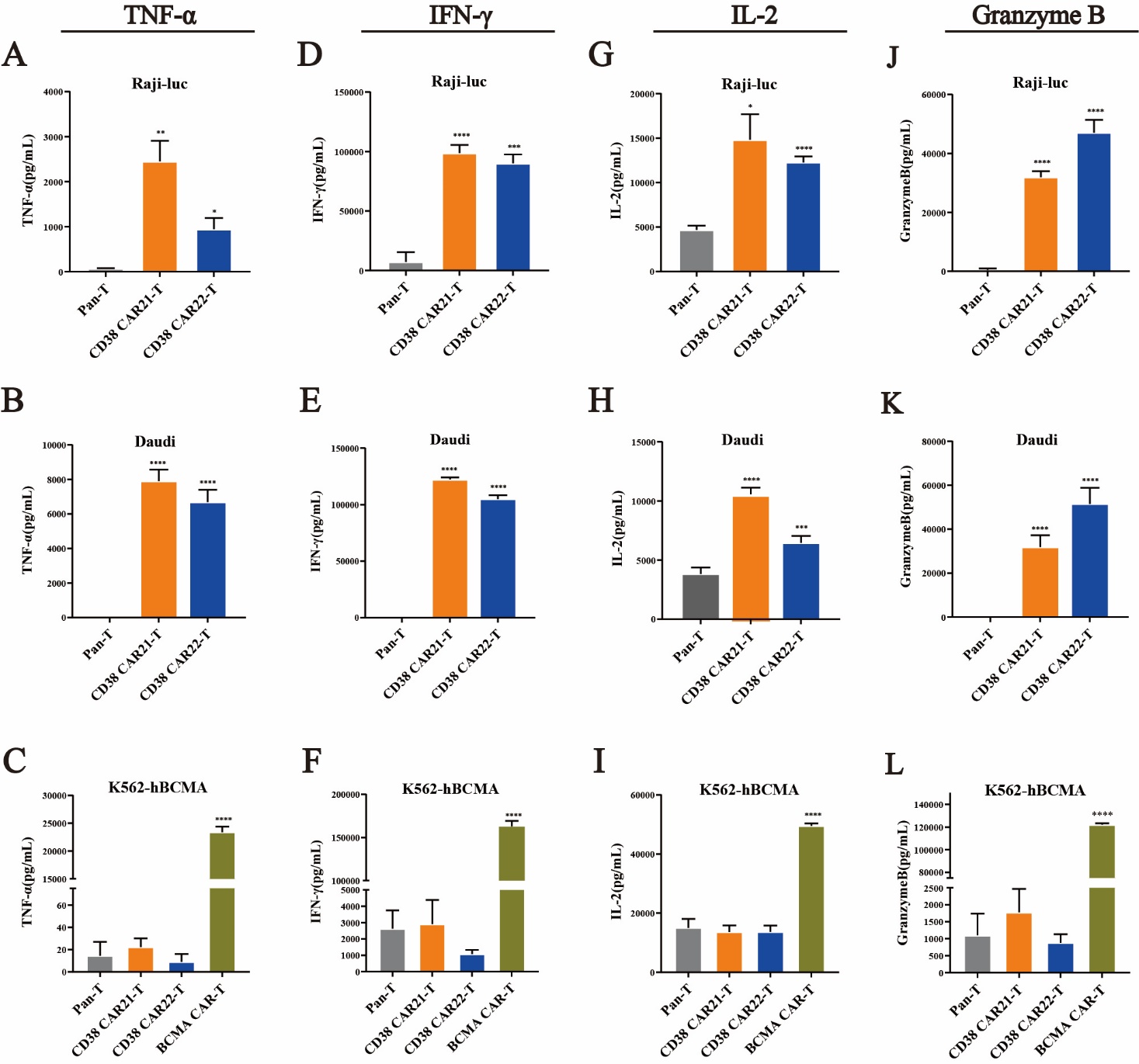
**

**Supplementary Figure 2. The secretion of cytokines by CD38 CAR-T cells.** ((A)-(L)) CD38 CAR-T cells or nontransduced T cells were cocultured with CD38-positive cells (Raji-luc, Daudi) or CD38-negative cells (K562-hBCMA) at an E:T ratio of 1:1 for 12 hours. The secretion of cytokines in the supernatant was measured by a CBA kit. ((A)-(C)) Concentrations of TNF-α; ((D)-(F)) Concentrations of IFN-γ. ((G)-(I)) Concentrations of IL-2; ((J)-(L)) Concentrations of granzyme B. The results are shown as the mean ± SD of three samples (n=3). * indicates *p* value <0.05; ** <0.01; *** <0.005 and ****<0.001, comparison of two groups was performed by Student’s t-test.


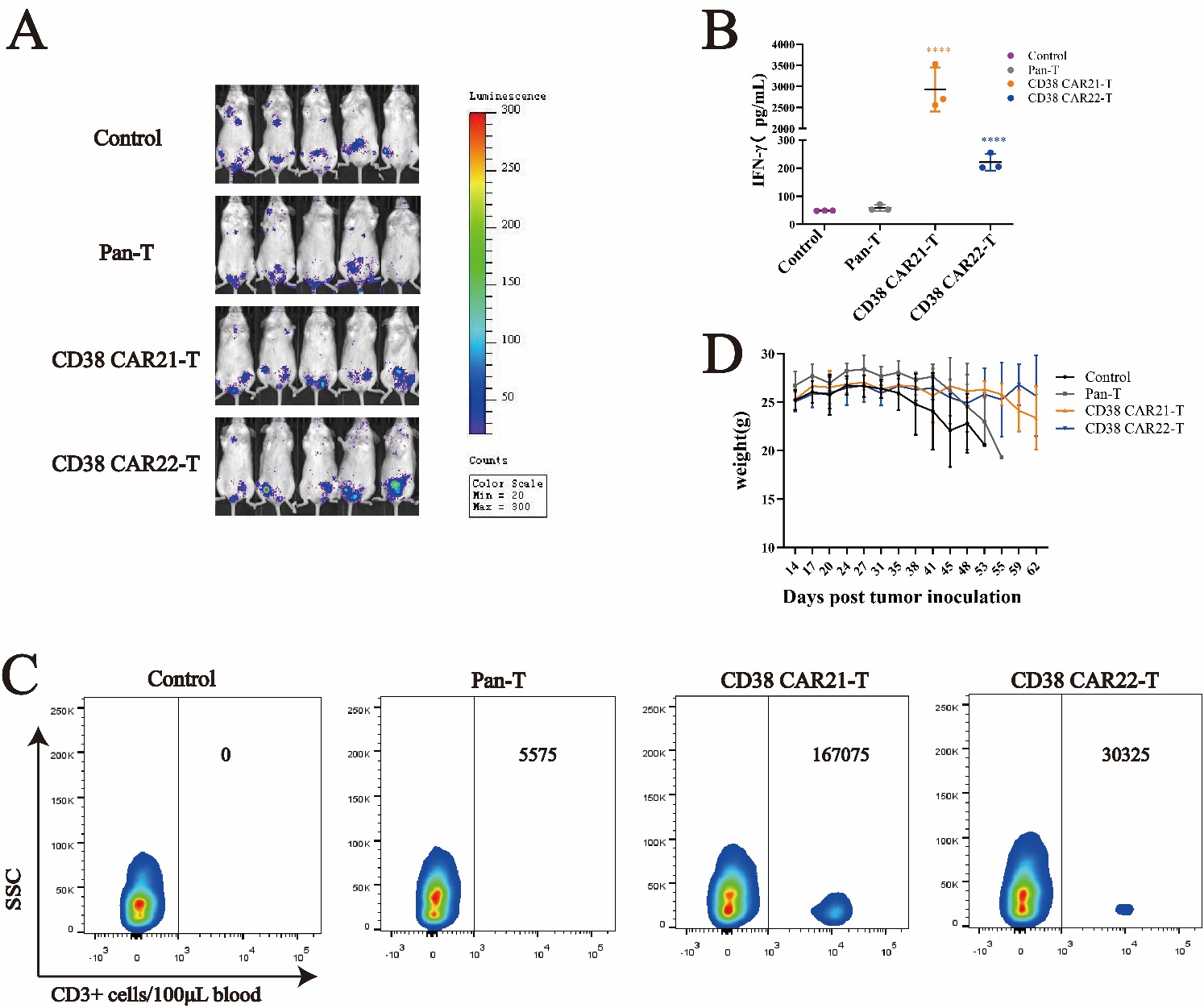


**Supplementary Figure 3.** **CD38 CAR-T cells exhibited significant antitumor activities in a mouse xenograft tumor model.** (A) Bioluminescence imaging of xenotransplanted NPG/Vst mice was performed on the 11th day, and then the mice were grouped randomly. (B) On the 16th day after tumor inoculation (the second day after the first injection of CAR-T cells), venous blood was collected to detect the secretion of the cytokine IFN-γ in plasma by a CBA kit (n=3); * indicates *p* value <0.05; ** <0.01; *** <0.005 and ****<0.001. Comparisons of two groups were performed by Student’s t-test. (C) On day 35 after tumor inoculation, the counts of injected CD3^+^ T cells per 100 μL peripheral blood of mice were measured by flow cytometry. The figure shows one representative experiment out of three. (D) Body weight of NPG/Vst mice after tumor inoculation (n=5).

**
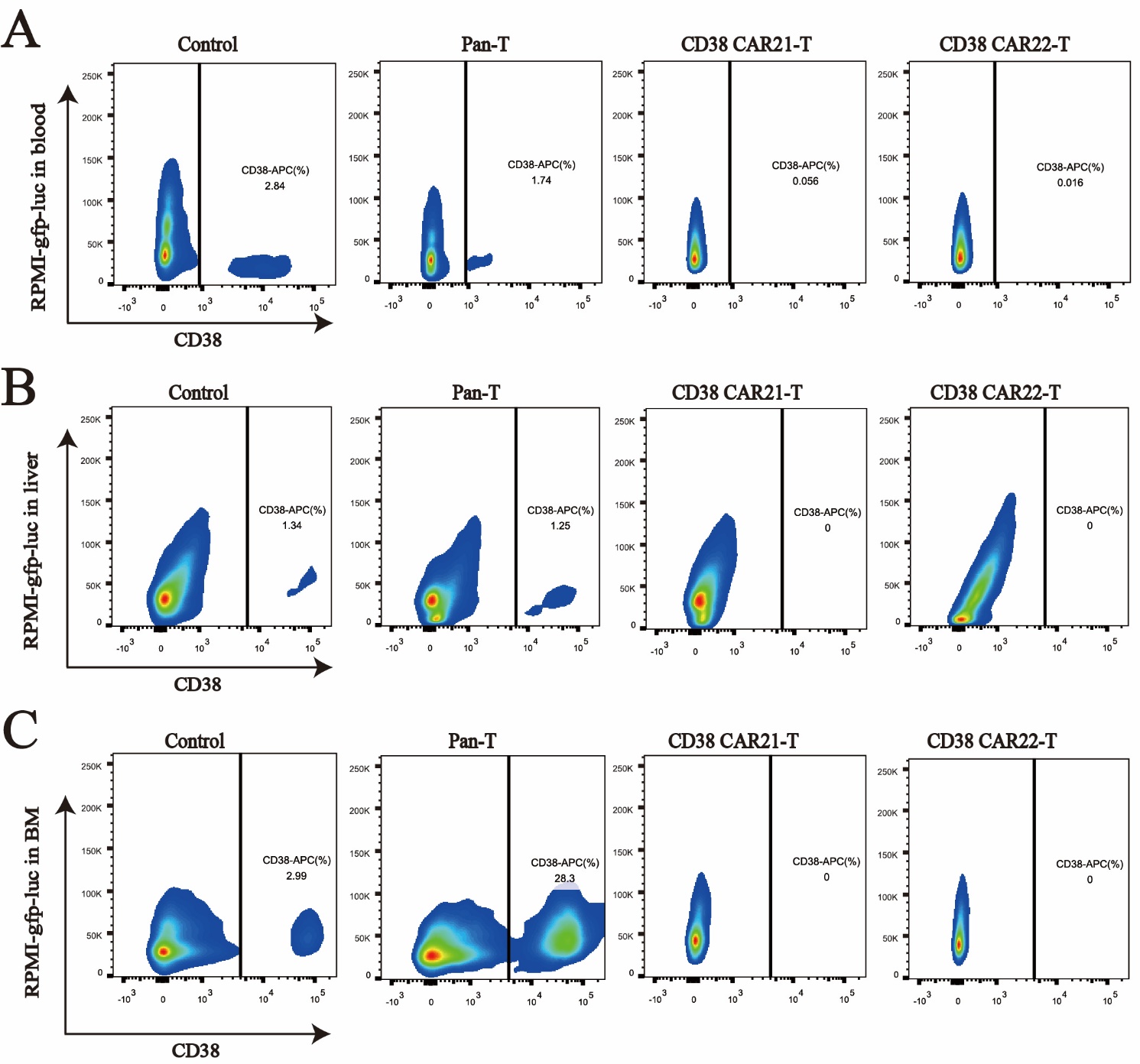
**

**Supplementary Figure 4. Residual detection of CD38 antigen in different tissues.** At the time of death of the mice, blood, liver, bone marrow and lysed erythrocytes were collected. The residual CD38-positive tumor cells were monitored by flow cytometry. (A) The percent of CD38 antigen in blood. (B) The percent of CD38 antigen in liver. (C) The percent of CD38 antigen in bone marrow(BM). The figure shown was one respresentative experiment.

**Supplemental Table 1. List of primer sequences for RT-qPCR.**

| SN | Gene Name | Sequence |
| --- | --- | --- |
| 1 | vector copy number primers | GACACCAGACTAAGAACCTAGAAC |
|  |  | CTCAAAGTAGACGGCATCGCAGCT |
| 2 | human glyceraldehyde-3-phosphate dehydrogenase (GAPDH) | CATGTTCGTCATGGGTGTGAACCA |
|  |  | ATGGCATGGACTGTGGTCATGAGT |
